# Gardenamide A Protects RGC-5 Cells from H_2_O_2_-Induced Oxidative Stress Insults by Activating PI3K/Akt/eNOS Signaling Pathway

**DOI:** 10.3390/ijms160922350

**Published:** 2015-09-15

**Authors:** Rikang Wang, Lizhi Peng, Jiaqiang Zhao, Laitao Zhang, Cuiping Guo, Wenhua Zheng, Heru Chen

**Affiliations:** 1 National Pharmaceutical Engineering Center for Solid Preparation in Chinese Herbal Medicine, Jiangxi University of Traditional Chinese Medicine, Nanchang 330006, China; E-Mail: wrk168ok@163.com; 2 Institute of Traditional Chinese Medicine and Natural Products, College of Pharmacy, Jinan University, Guangzhou 510632, China; E-Mails: 15900088863@163.com (L.P.); jqzhaodzs@163.com (J.Z.); zhanglaitao25@sina.com (L.Z.); guocui555@163.com (C.G.); 3 Faculty of Health Sciences, University of Macao, Macao, China; 4 Guangdong Province Key Laboratory of Pharmacodynamic Constituents of TCM and New Drugs Research, Guangzhou 510632, China

**Keywords:** gardenamide A, oxidative stress, cell apoptosis, neuroprotection, neurotoxicity

## Abstract

Gardenamide A (GA) protects the rat retinal ganglion (RGC-5) cells against cell apoptosis induced by H_2_O_2_. The protective effect of GA was completely abrogated by the specific phosphoinositide 3-kinase (PI3K) inhibitor LY294002, and the specific protein kinase B (Akt) inhibitor Akt VIII respectively, indicating that the protective mechanism of GA is mediated by the PI3K/Akt signaling pathway. The specific extracellular signal-regulated kinase (ERK1/2) inhibitor PD98059 could not block the neuroprotection of GA. GA attenuated the levels of reactive oxygen species (ROS) and malondialdehyde (MDA) induced by H_2_O_2_. Western blotting showed that GA promoted the phosphorylation of ERK1/2, Akt and endothelial nitric oxide synthase (eNOS), respectively, and effectively reversed the H_2_O_2_-inhibited phosphorylation of these three proteins. LY294002 completely inhibited the GA-activated phosphorylation of Akt, while only partially inhibiting eNOS. This evidence implies that eNOS may be activated directly by GA. PD98059 attenuated only partially the GA-induced phosphorylation of ERK1/2 with/without the presence of H_2_O_2_, indicating that GA may activate ERK1/2 directly. All these results put together confirm that GA protects RGC-5 cells from H_2_O_2_ insults via the activation of PI3K/Akt/eNOS signaling pathway. Whether the ERK1/2 signaling pathway is involved requires further investigations.

## 1. Introduction

It is well recognized nowadays that oxidative stress is closely related to the development of neuronal diseases such as Alzheimer’s disease (AD), Parkinson’s disease (PD), and ischemic and hemorrhagic stroke [1,2]. There is strong evidence linking oxidative stress to the pathology of retinal diseases including retinitis pigmentosa (RP), age related macular degeneration (AMD) and retinal detachment [3,4,5]. Oxidative stress does not exhibit a specific clinical symptom, however, it may be mediated by reactive oxygen species (ROS) or/and reactive nitrogen species (RNS). Conventionally, hydrogen peroxide (H_2_O_2_) is used to induce oxidative stress and/or endoplasmic reticulum (ER) stress in cells [6,7]. A promising strategy to attenuate oxidative stress insults is to apply antioxidants in the treatment of both acute and chronic neurodegenerative diseases [8,9,10].

**Figure 1:**
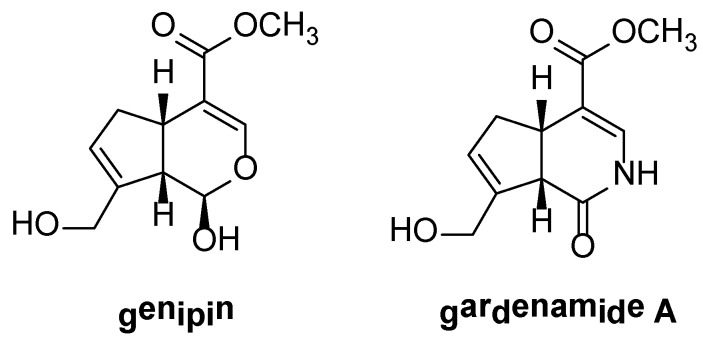
Chemical structures of genipin and gardenamide A.

Previously, we developed a stable genipin derivative gardenamide A (GA) (Figure 1), which was also found in *Rothmannia urcelliformis* that is widespread in the forests of East Africa, and in the fruit of *Gardenia jasminoides* [11]. Like genipin, GA protects PC12 cells from toxicities induced by 6-hydroxydopamine and serum deprivation, respectively, with higher activity [12]. It is likely that GA can play a role as antioxidant. Therefore, we would like to determine whether GA could protect neuronal cells from oxidative stress insults induced by H_2_O_2_ and the mechanism(s) involved.

The protein kinase B (Akt) is a survival kinase and a main downstream target of the phosphoinositide 3-kinase (PI3K). Growth factors and hormones promote the survival of a variety of cells by stimulating the PI3K/Akt pathway [13]. Active Akt phosphorylates its substrates including Forkhead box protein (FOX) transcription factors, Bcl-2-associated death promoter (Bad) and endothelial nitric oxide synthase (eNOS) [14,15,16]. The phosphorylation of eNOS at Ser1177 causes the activation of this enzyme and the increase in the production of nitric oxide (NO) in target tissues. The diffusible messenger molecule NO is an important mediator of survival and death in many cell types. Physiological concentration of NO avidly scavenges superoxide anion, preventing superoxide anion from forming its dismutation product H_2_O_2_, and promoting cell survival [17,18,19]. By inducing eNOS activity, activation of the PI3K/Akt pathway can enhance the cell survival [17,20].

Although the rat retinal ganglion (RGC-5) cell line is believed to be not of retinal ganglion cell origin, it still represents the retinal neuronal precursor cells and hence is appropriate for biochemical studies in the neuronal cells. Therefore, in this study, we evaluated the effects of GA on H_2_O_2_-induced apoptosis of RGC-5 cells. Its underlying mechanisms have also been investigated. Our results show that GA protects RGC-5 cells from apoptosis induced by H_2_O_2_ by the activation of PI3K/Akt/eNOS signaling pathways and the regulation of reactive oxygen species (ROS)/malondialdehyde (MDA).

## 2. Results and Discussion

### 2.1. GA Dose-Dependently Protected RGC-5 Cells from H_2_O_2_-Induced Insults

**Figure 2:**
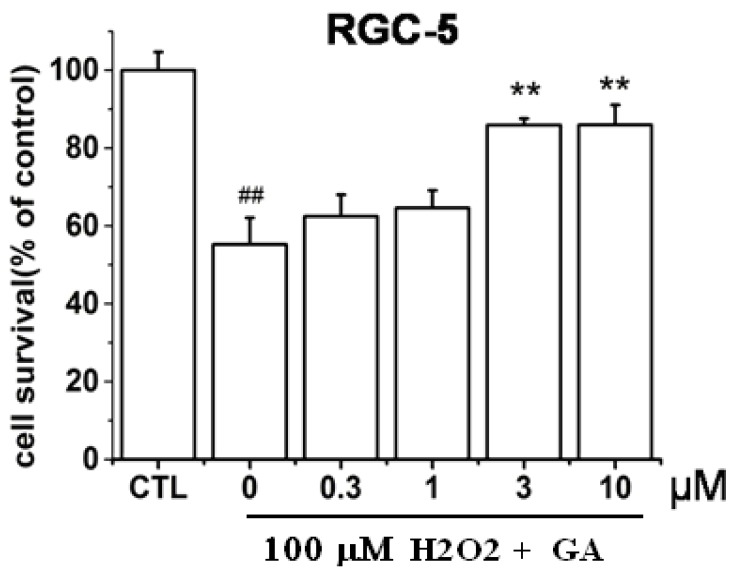
Protective effects of GA on RGC-5 cells death induced by H_2_O_2_. Cells were treated with different concentrations of GA and were exposed to 100 µM H_2_O_2_. The cell viability was determined by MTT assay. ^##^ *p* < 0.01 *vs.* control; ** *p* < 0.01 *vs.* model (*n* = 3).

By using MTT assay to determine the cell viability, it was found that treatment of 100 µM H_2_O_2_ to RGC-5 cells for 24 h caused about 48% ± 1.6% cell death (Figure 2). However, pre-treatment of GA for 2 h protected RGC-5 cells from insults induced by H_2_O_2_ in a concentration-dependent manner (Figure 2). Statistically significant inhibition effect of GA commenced at 3 μM.

### 2.2. GA Protected RGC-5 Cells against Apoptosis Induced by H_2_O_2_

**Figure 3:**
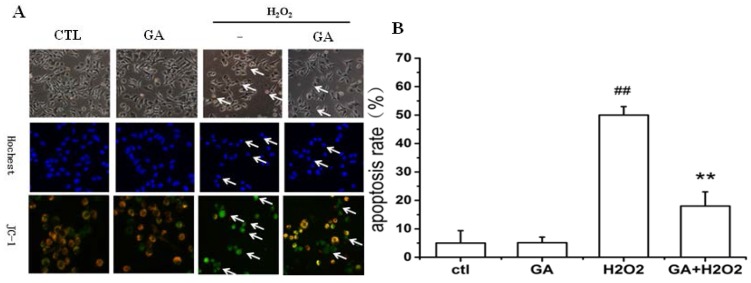
GA inhibited the apoptosis of RGC-5 cells induced by H_2_O_2_. RGC-5 cells were pre-incubated with or without GA (10 μM) for 2 h before the addition of 100 μM H_2_O_2_. Cells were incubated with Hoechst 33,258 staining. (**A**) Morphological changes shown by fluorescence microscope (200×) image analysis; (**B**) Histogram showing the apoptosis rate of RGC-5 cells after H_2_O_2_ exposure in the presence or absence of GA compared to H_2_O_2_-treated control group. The results shown were from a representative experiment, which was repeated at least three times. ^##^ *p* < 0.01 *vs.* control group; ** *p* < 0.01 *vs.* H_2_O_2_-treated group. Arrows in the first row pointed to represented cells with morphological changes; arrows in the second row pointed to represented apoptotic cells without blue color; arrows in the third row pointed to represented apoptotic cells with green fluoresecence.

It was clearly demonstrated in Figure 3 that treatment of 100 μM H_2_O_2_ to RGC-5 cells for 24 h caused abnormal change of cell morphology, nuclear chromatin condensation (Figure 3A, first row), and cell apoptosis (Figure 3A, second row). The cell apoptosis rate was 50.4% ± 3.6% (Figure 3B). Quite interestingly, cells pretreated with GA at a dose of 10 μM displayed improved morphology and suppressive nuclear condensation (Figure 3A, first row). Pre-treatment of cells with 10 µM GA significantly prevented the decline of mitochondrial membrane potential induced by H_2_O_2_ (Figure 3A, third row). The cell apoptosis rate was significantly decreased from 50.4% ± 3.6% to 26.4% ± 4.3% (Figure 3B).

### 2.3. The Protective Effect of GA on RGC-5 Cells Was Mediated by Endothelial Nitrioxide Synthase (eNOS)

**Figure 4:**
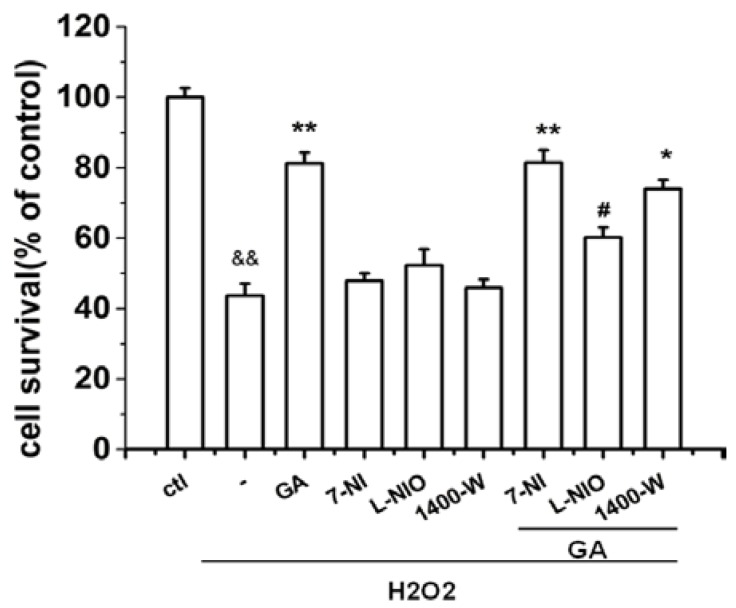
GA promoted the survival of RGC-5 cells by endothelial nitric oxide synthase (eNOS). RGC-5 cells preincubated with various NOS subtype inhibitors were treated with H_2_O_2_ (100 µM) in the absence or presence of GA (10 μM). The cell viability was determined by MTT assay. Only L-NIO attenuated significantly the protective effect of GA. ^&&^ *p* < 0.01 *vs.* control group; * *p* < 0.05, ** *p* < 0.01 *vs.* H_2_O_2_-treated group; ^#^ *p* < 0.05 *vs.* GA + H_2_O_2_ treated group (*n* = 3).

The activation of neuronal NO synthase (nNOS) is one of the major protective mechanisms of genipin [21]. To determine whether this mechanism is also involved in the effect of GA, we examined the role of various NO synthase subtypes in the protective effect of GA in H_2_O_2_-impaired RGC-5 cells. As indicated in Figure 4, the protective effect of GA on RGC-5 cells was significantly inhibited by L-NIO, a specific eNOS inhibitor. These results clearly indicated that eNOS was involved in the neuroprotective effect of GA in RGC-5 cells. Surprisingly, 7-NI, a specific nNOS inhibitor had no inhibition effect; while 1400W, a specific iNOS, had weak inhibition effect.

To further evaluate the effect of various NOS subtypes, the activity of each NOS subtype was determined by a Typed Nitric Oxide Synthase (NOS) Detection Kit. As shown in Table 1, compared to the control group, H_2_O_2_ significantly inhibited the activities of total NOS (tNOS), constitutive NOS (cNOS), and eNOS; while stimulated the activity of inducible NOS (iNOS). On the contrary, treatment of GA alone stimulated the activities of tNOS, cNOS, and eNOS, and attenuated the activity of iNOS. As indicated in Table 1 row 5 *vs.* 4, pretreatment of GA reversed the effects of H_2_O_2_ on each type of NOS.

**Table 1:** Effects of GA, H_2_O_2_ and GA/H_2_O_2_ on the NOS activities in RGC-5 cells.

Groups	tNOS (U/mL)	cNOS (U/mL)	eNOS (U/mL)	iNOS (U/mL)
ctrl	8.23 ± 0.43	6.42 ± 0.64	3.32 ± 0.35	1.81 ± 0.33
GA	9.52 ± 0.34	8.36 ± 0.52 ^&&^	4.26 ± 0.22 ^&&^	1.16 ± 0.22
H_2_O_2_	5.45 ± 0.22 **	1.82 ± 0.19 **	1.03 ± 0.17 **	3.63 ± 0.42 **
H_2_O_2_ + GA	7.51 ± 0.58 ^##^	6.20 ± 0.28 ^##^	3.14 ± 0.23 ^##^	1.31 ± 0.21 ^##^

RGC-5 cells were treated with GA, H_2_O_2_ and their mixture, respectively. Then the activities of tNOS (total NOS), cNOS (constitutive NOS), eNOS (endothelial NOS) and iNOS (inducible NOS) were tested by Typed Nitric Oxide Synthase (NOS) Detection Kit. H_2_O_2_ inhibited tNOS and cNOS while stimulated iNOS activities. On the contrary, GA activated tNOS and cNOS, and inhibited iNOS. GA reversed the effect of H_2_O_2_. U/mL: 1 nmol of NO formed/mL cell lysates in one minute. Difference is considered significant at ** *p* < 0.01, ^&&^ *p* < 0.01 *vs.* control group; ^##^ *p* < 0.01 *vs.* H_2_O_2_-treated group. All values are expressed as mean ± SD (*n* = 3).

### 2.4. GA Time- and Dose-Dependently Stimulated the Phosphorylation of ERK1/2 and Akt in RGC-5 Cells

Both ERK1/2 and Akt pathways are the two main pathways involved in mediating the survival and many growth factors in a variety of cell types [13,22,23]. The activation/phosphorylation of ERK1/2 and Akt proteins modulate the activation of eNOS in various tissues. To determine the roles of these two pathways in the activation of eNOS induced by GA and their role in GA protective effects, we first examined the phosphorylation of Akt/eNOS/ERK1/2 stimulated by GA using Western blotting with corresponding phospho-antibodies.

**Figure 5:**
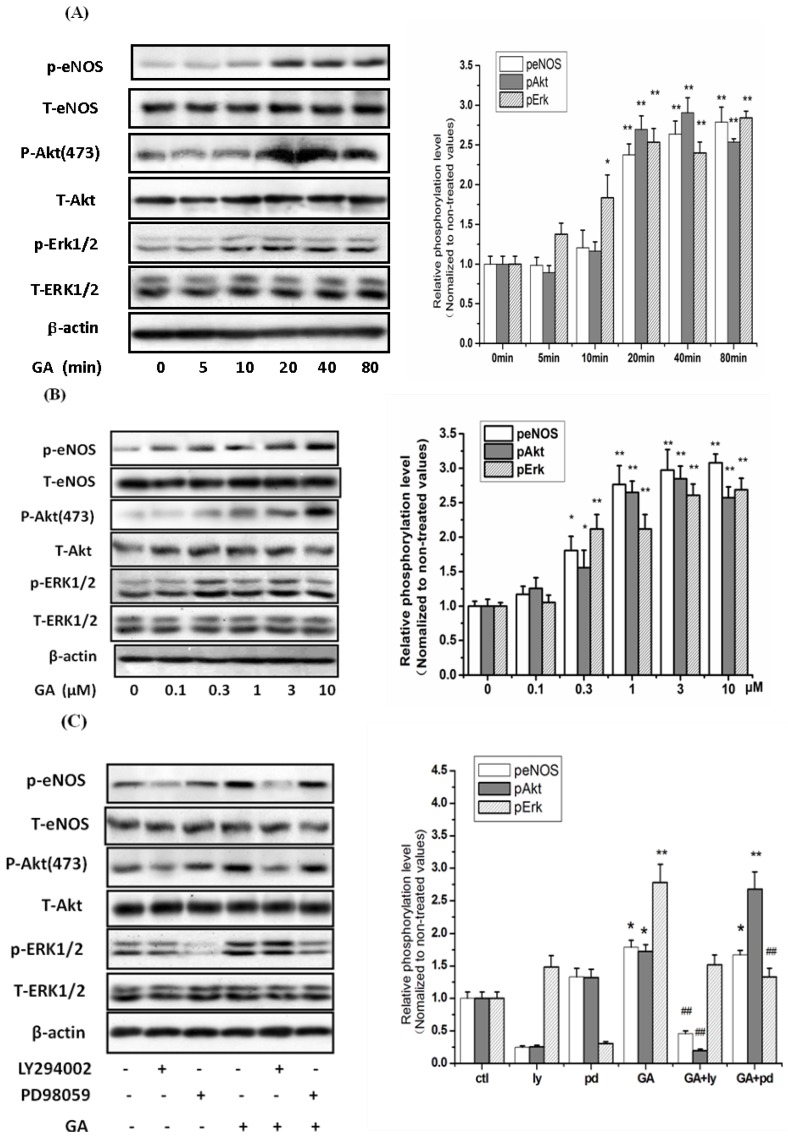
GA activated the phosphorylation of eNOS, Akt and ERK1/2. RGC-5 cells were treated with 10 μM GA for 5–80 min or 0.1–10 μM GA for 20 min. The phosphorylation of eNOS, Akt and ERK1/2 were determined by Western blotting with anti-phospho-Akt (Ser473), anti-phospho-ERK1/2, anti-phospho-eNOS (Ser1177), respectively, using β-actin as control. GA time-dependently induced the phosphorylation of Akt, eNOS, and ERK1/2 in RGC-5 cells. (**A**) Time course; (**B**) Dose course; (**C**) The effects of pathway inhibitors on the GA-activated phosphorylation of Akt, eNOS and ERK1/2. Results represent prototypical experiments replicated at least three times. * *p* < 0.05, ** *p* < 0.01, ^&&^ *p* < 0.01 *vs.* control group; ^##^ *p* < 0.01 *vs.* GA group.

As shown in Figure 5, GA time- and dose-dependently stimulated the phosphorylation of eNOS, Akt and ERK1/2. The phosphorylation of Akt increased significantly at about 20 min and peaked at 40 min; for ERK1/2, it increased significantly at 10 min and peaked after 80 min; while the phosphorylation of eNOS significantly increased at 20 min and peaked after 80 min (Figure 5A). A significant increase of the phosphorylation level of Akt began at the concentration of 0.3 µM, peaked at 3 µM after 20-min duration; as indicated in Figure 5B, the phosphorylation level of eNOS and ERK1/2 significantly increased at dose of 0.3 µM; while the phosphorylation level of eNOS and ERK1/2 did not peak at the maximal range of the current dose. It was found that pre-incubation of PI3K inhibitor LY294002 (10 µM) completely abrogated the GA-induced phosphorylation of Akt, but only partially to that of eNOS; while the ERK1/2 inhibitor PD98059 (30 µM) only partially abrogated the GA-induced phosphorylation of ERK1/2 (Figure 5C).

### 2.5. Neuroprotective Action of GA against H_2_O_2_-Induced Impairments to RGC-5 Cells Was Mediated by the Activation of the PI3K/Akt Signaling Pathway

**Figure 6:**
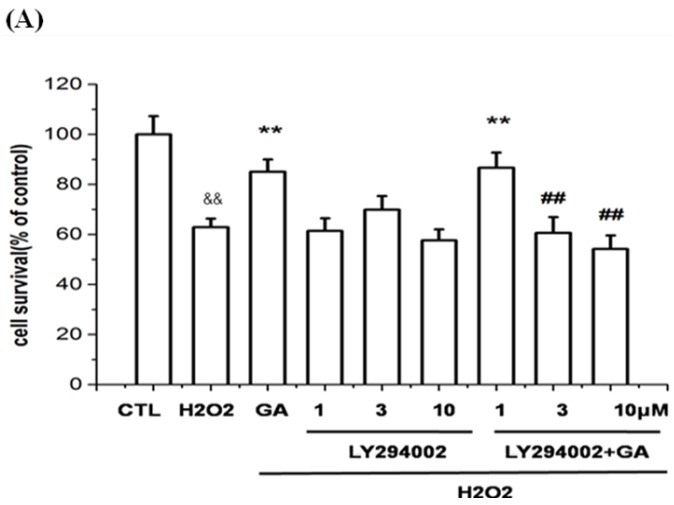

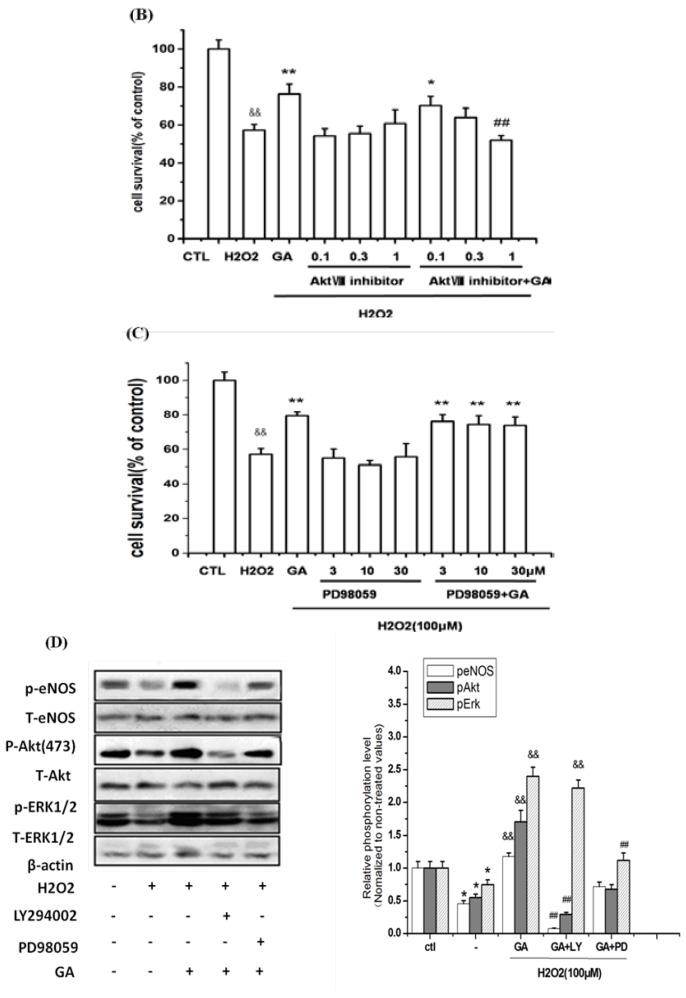
PI3K inhibitor LY294002, and Akt inhibitor Akt inhibitor VIII, respectively, blocked the effect of GA on RGC-5 cells. Cells pretreated with the PI3K inhibitor LY294002, Akt inhibitor Akt inhibitor VIII, and MEK inhibitor PD98059, respectively, were treated with H_2_O_2_ in the absence or presence of GA (10 μM). The cell viability and the phosphorylation levels of Akt, eNOS and ERK1/2 were determined. (**A**) PI3K inhibitor LY294002 dose- dependently blocked the protective effect of GA on RGC-5 cells; (**B**) Akt inhibitor Akt VIII dose-dependently blocked the effect of GA on RGC-5 cells; (**C**) MEK inhibitor PD98059 showed no effect; (**D**) H_2_O_2_ blocked the phosphorylation of Akt, eNOS and ERK1/2, while GA reversed the blockade. LY294002 completely inhibited the GA-activated phosphorylation of Akt and eNOS. PD98059 partially inhibited the GA-activated phosphorylation of ERK1/2 in a statistically significant manner, while it partially inhibited the GA-activated phosphorylation of Akt and eNOS in a non-statistically significant manner. Results were expressed as the percentage of the corresponding control value, which was set at 100%. Data are shown as the mean ± SEM. and representative assays from at least three independent experiments. ^&&^ *p* < 0.05 *vs.* control group, * *p* < 0.05, ** *p* < 0.01 *vs.* H_2_O_2_-treated group; ^##^ *p* < 0.05 *vs.* GA-treated group.

It was clearly shown in Figure 6A,B that the neuroprotective effects of GA against H_2_O_2_-induced impairments in RGC-5 cells were reversed by LY294002, and Akt inhibitor VIII (an Akt specific inhibitor), respectively, in a dose-dependent manner. However, the involvement of PD98059 did not attenuate the GA protection (Figure 6C). These results were inconsistent with that from Western blotting. In Figure 6D, it is shown that H_2_O_2_ decreased the phosphorylation of Akt, eNOS and ERK1/2 (*lane* 2 *vs. lane* 1); while GA reversed the inhibition effects of H_2_O_2_ (*lane* 3 *vs*. *lane* 2). The effect of GA on the phosphorylation of Akt and eNOS was inhibited significantly by the pre-incubation of LY294002 (*lane* 4 *vs. lane* 3); Surprisingly, PD98059 only partially inhibited the GA-activated phosphorylation of ERK1/2 in a statistically significant manner while it partially inhibited the GA-activated phosphorylation of Akt and eNOS in a non-statistically significant manner (*lane* 5 *vs. lane* 3).

### 2.6. GA Blocked ROS Production and Lipid Peroxidation Induced by H_2_O_2_ in RGC-5 Cells

**Figure 7:**
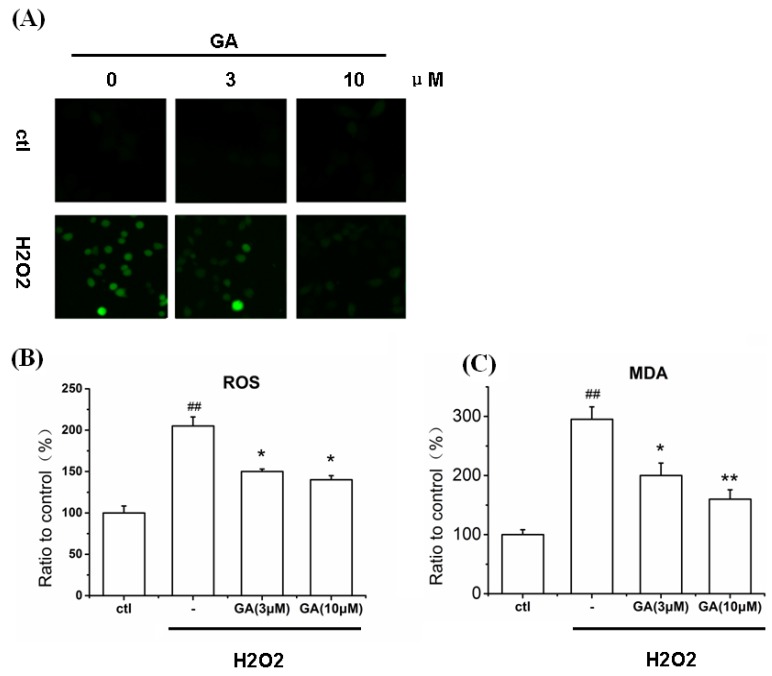
Attenuation of H_2_O_2_-induced ROS and MDA production by GA. Cells pretreated with/without GA (3 and 10 μM, respectively) for 30 min, were exposed to H_2_O_2_ (100 μM) for 12 h. The production of ROS and MDA were then determined. (**A**) The fluorescence intensity of DCFH-DA, which were photographed using high content screening system; (**B**) Histogram showing the ROS levels in RGC-5 cells after treatment with H_2_O_2_ in the presence or absence of GA; (**C**) Histogram showing the MDA levels in RGC-5 cells after treatment with H_2_O_2_ in the presence or absence of GA. ^##^ *p* < 0.01 *vs.* control group; * *p* < 0.05, ** *p* < 0.05 *vs.* H_2_O_2_-treated group (*n* = 3).

Previously, it was shown that the toxicity of H_2_O_2_ was mediated through the production of ROS and that genipin could quench the ROS production [24,25]. Therefore, we studied the effect of GA on H_2_O_2_-induced productions of ROS and malondialdehyde (MDA) in RGC-5 cells. It must be pointed out that the level of ROS determined by the current assay included the H_2_O_2_ residues inside the cells. Nevertheless, in Figure 7A–C, it was displayed that H_2_O_2_ increased the levels of ROS and MDA; while treatment of GA at a dose of 3 and 10 μM, respectively, decreased the production of ROS and MDA induced by H_2_O_2_. The levels of ROS generation dropped from 210% to 148%; while the levels of MDA generation lowered from 300% to 200% (3 µM), and 150% (10 µM), respectively.

### 2.7. Discussion

In this study, we examined the effects of GA on RGC-5 cell insults induced by H_2_O_2_ and the cellular signaling mechanisms involved. Our results showed that GA dose-dependently decreased RGC-5 cells apoptosis induced by H_2_O_2_. The effect of GA was abolished by the PI3K inhibitor, the Akt inhibitor, and the eNOS inhibitor, respectively; while the inhibitor of ERK1/2 pathway has no effect. This indicated that only the PI3K/Akt/eNOS pathway was involved in the neuroprotection of GA.

Substantial evidences have displayed that oxidative stress plays a critical role in the pathogenesis of retinal diseases like AMD or RP [26,27]. For example, in the retina, elevated levels of ROS disrupted mitochondrial function of retinal pigment epithelium cells leading to the apoptotic cell death, which eventually resulted in the death of photoreceptor cells [28]. Therefore, the inhibitory mediators of ROS such as antioxidants could be beneficial for therapeutic prevention of retinal diseases and other neurodegenerative disorders.

GA has been shown the effects to protect PC12 cells against serum-deprivation insults [12]. In the present investigation, it was clearly displayed that H_2_O_2_ concentration dependently caused the apoptosis of RGC-5 cells and the increase of ROS and MDA, which are closely related to oxidative stress. Interestingly, our results from both MTT and Hoechst staining assays showed that GA protected RGC-5 cells from apoptosis caused by oxidative stress in a dose-dependent manner.

#### 2.7.1. GA Decreased ROS and MDA Levels in RGC-5 Cells Induced by H_2_O_2_

Oxidative stress is an imbalance between pro-oxidant like ROS and antioxidant systems. An increase in pro-oxidant status results in oxidative damage to essential biomolecules like proteins and lipids, and changes the biological functions of these molecules. Our results showed that H_2_O_2_ increased the production of ROS in RGC-5 cells. This is consistent with a previous report which showed that the toxicity of H_2_O_2_ was mediated through the production of ROS [25]. Fortunately, GA showed the effect to eliminate significantly the level of ROS and protect RGC-5 cells from oxidative stress impairments. As expected, GA can be applied as an antioxidant as can its parent compound genipin [25].

Pathological levels of ROS affect mitochondrial membrane, which is an intracellular process contributing to apoptosis [29]. In the current research, GA attenuated accumulation of intracellular ROS in RGC-5 cells. In addition, we observed that H_2_O_2_ insult was followed by loss of the mitochondrial membrane potential. Fortunately, treatment with GA significantly reversed this process. As a matter of fact, the loss of the mitochondrial membrane potential may result in mitochondrial dysfunction, which appears to be a widespread feature in both sporadic and inherited forms of PD [30,31,32].

As we know, in the central nervous system, peroxidation of lipids is the key mechanism of the damage resulting from the action of free radicals. Lipid peroxidation of unsaturated fatty acids produces high levels of MDA and this can be a marker of oxidative damage. It was demonstrated that H_2_O_2_ increased the production of MDA in RGC-5 cells and GA significantly reversed this effect.

However, mechanisms underlying these effects of GA on ROS and MDA in RGC-5 are not clear at the current state. It is possible that GA inhibits the production of ROS and MDA by the induction of antioxidant genes. In accordance with this hypothesis, genipin, the parent compound of GA, was reported to block the increase of ROS induced by TNF-α through the activation of heme oxygenase-1 (HO-1) [25]. We also found that the genipin derivative CHR21 attenuated the sodium nitroprusside (SNP)-caused ROS level by increasing the activities of two antioxidative proteins, the glutamate-cysteine ligase catalytic subunit (GCLC) and superoxide dismutase 1 (SOD1) [33].

#### 2.7.2. GA Promoted Survival of RGC-5 by Activating eNOS

Several reports have shown that nitric oxide synthase (NOS)/nitric oxide (NO) are indeed involved in the neuroprotective effects of genipin and its derivatives [12,34,35,36]. Here, we found that GA increased the level of tNOS, cNOS, and eNOS, and reversed the effects of H_2_O_2_ to each type of NOSs. The eNOS specific inhibitor L-NIO significantly blocked the neuroprotective effect of GA on the survival of RGC-5 cells but not completely. These results implied the involvement of eNOS in the protection of GA against H_2_O_2_-caused insults in RGC-5 cells. However, it was found that nNOS was not involved in this neuroprotective process. Although in the other case, nNOS was found involved in the protection of 6-hydroxydopamine (6-HODA)-induced impairments in PC12 cells [12]. Is this because of the cell insults caused by different agents or because of the different cell lines used? This requires further studies.

iNOS is involved in immune response, binds calmodulin at physiologically relevant concentrations, and produces NO as an immune defense mechanism. An oxidative environment may induce the high-output of iNOS. High levels of NO have the opportunity to react with superoxide leading to peroxynitrite formation and cell toxicity. It was disclosed that H_2_O_2_ caused the increase of iNOS, while GA inhibited the activity of iNOS. The iNOS inhibitor 1400W displayed a weak inhibition against GA protection to RGC-5 cells insults induced by H_2_O_2_.

#### 2.7.3. GA Promoted Survival of RGC-5 by Activation of the PI3K/Akt/eNOS Signaling Pathway

The PI3K/Akt pathway is an important survival pathway against several cytotoxins including oxidative stress [14,37]. It was reported that genipin and some of its derivatives can activate the PI3K/Akt pathway. For example, genipin activated the PI3K/Akt pathway by increasing the phosphorylation of insulin receptor substrate-1 (IRS-1) in C2C12 myotubes [38]. As we know, Akt is an upstream kinase of eNOS. Phosphorylation of Akt lead to the activation of eNOS [15]. Therefore, GA may probably activate eNOS by stimulating the PI3K/Akt signaling pathway. This hypothesis is supported by the following evidences. Firstly, GA time- and concentration-dependently stimulated the phosphorylation of Akt and eNOS in RGC-5 cells. Secondly, either PI3K specific inhibitor LY292002 or Akt inhibitor VIII, respectively, blocked the phosphorylation of eNOS and Akt. At last, GA significantly reversed the H_2_O_2_-induced inhibition against the phosphorylation of Akt and eNOS. All these data support the proposition that GA promotes the survival of RGC-5 cells from H_2_O_2_-induced injury via the PI3K/Akt/eNOS pathway. Interestingly, LY292002 abrogated only partially the GA-induced phosphorylation of eNOS. This evidence implied that the phosphorylation of eNOS occurred not only from the activation of Akt but also from the direct activation of GA.

The importance of the ERK1/2 pathway in mediating apoptosis caused by various other stimuli is well established [39,40]. It was reported that genipin promoted the survival of PC12h cells by activating the ERK1/2 pathway [41,42]. In the current study, it has been shown that GA time- and concentration-dependently stimulated the phosphorylation of ERK1/2. Surprisingly, the ERK1/2 specific inhibitor PD98059 blocked only partially the GA-induced phosphorylation of ERK1/2 without the presence of H_2_O_2_ (Figure 5C). However, it did completely abrogate the phosphorylation of ERK1/2 without the presence of GA and H_2_O_2_. It seemed that the phosphorylation of ERK1/2 was not completely from the activation of mitogen-activated protein kinase kinase (MEK), but partially from the direct activation of GA.

GA no doubt activated the H_2_O_2_-inhibited phosphorylation of ERK1/2. However, as the specific ERK1/2 inhibitor, PD98059 could only partially abrogate the GA-activated phosphorylation of ERK1/2 at the presence of H_2_O_2_ (Figure 5D). Interestingly, PD98059 seemed to block partially the phosphorylation of Akt and eNOS with the presence of H_2_O_2_. In the absence of H_2_O_2_, PD98059 showed no inhibition to the phosphorylation of Akt and eNOS (Figure 4C). The exact mechanism is not clear at this moment. It is speculated that PD98059 may be oxidized by H_2_O_2_ and turned into a new agent. PD98059 could not inhibit the protective effect of GA as LY292002 did (Figure 5C). These data put together cannot exclude the involvement of ERK1/2 pathway in the neuroprotection of GA against H_2_O_2_-inuced RGC-5 cell insults. Further investigations are required to make this clear.

## 3. Experimental Section

### 3.1. Chemicals and Reagents

GA was synthesized as described before [12]. RGC-5 cells were purchased from the Centre of Cells Resource, Shanghai Institute of Life Science, Chinese Academy of Sciences, China. H_2_O_2_ were purchased from Research Biochemicals International (St. Louis, MO, USA); Fetal bovine serum (FBS) and RPMI-1640 medium were purchased from Gibco-BRL (Grand Island, NY, USA); A014 Typed Nitric Oxide Synthase (NOS) Detection Kit was from Nanjing Jiancheng Institute of Bioengineering (Nanjing, China); Anti-β-actin antibody, 3-(4,5-dimethylthiazol-2-yl)-2,5-diphenyl tetrazolium bromide (MTT), poly-d-lysine and dimethylsulfone (DMSO) were from Sigma–Aldrich (Shanghai, China); 7-Nitroindazole (7-NI), *N*-[3-(aminomethyl)benzyl]acetamidine dihydrochloride (1400W) and N5-(1-imino-3-butenyl)-l-ornithine (L-NIO) were from Santa Cluz Biotech (Santa Cluz, CA, USA); Hoechst 33258, MDA detection kit, BCA protein assay kit were from Beyotime Institute of Biotechnology (Haimen, China); Anti-phospho-Akt (Ser473), and phospho-ERK1/2 antibodies were purchased from Cell Signaling Technology (Woburn, MA, USA); Anti-phospho-eNOS (Ser1177) antibody was purchased from Signalway Antibody (College Park, MD, USA), PI3-K inhibitor LY294002, Akt inhibitor VIII and PD98059 were obtained from Calbiochem (Temecula, CA, USA).

### 3.2. Cell Culture

RGC-5 cells were cultured in RPMI-1640 medium containing 10% FBS. RGC-5 cells were passaged by every 3–4 days using streptomycin (100 μg/mL) and penicillin (100 U/mL), and incubated at 37 °C with 5% CO_2_ humidified atmosphere. Cultured media were replaced with fresh RPMI-1640 twice a week. Stock culture was routinely subcultured at 1:5 ratio weekly.

### 3.3. MTT Assay

Cell viability was estimated using an MTT assay. In short, after 24 h treatments, the culture medium was removed and replaced with 90 µL of fresh DMEM. Ten microliters of 5 mg/mL MTT in phosphate-buffered saline (PBS) was added to each well and the plates were incubated at 37 °C for another 3 h. Then, supernatants were discarded. Afterwards, DMSO (100 µL) was added to each well and the solutions were mixed thoroughly. Then, the plates were incubated at 37 °C for another 10 min. Each sample was mixed again and the resultant formazan was measured at 570 nm using a BIO-RAD680 plate reader (Thermo, Walsam, MA, USA). The experiments were repeated at least three times and compared with the control experiment.

### 3.4. Detection of Apoptosis by Hoechst Staining

The cells grown on slides were treated with GA and/or H_2_O_2_. After washing with PBS, the cells were stained with Hoechst 33258 (5 μg/mL) for 10 min at 37 °C. Then, Hoechst 33258 was removed by washing cells with PBS and the rate of apoptosis was calculated by a high content screening system (ArrayScanVTI, Thermo Fisher Scientific, Walsam, MA, USA).

### 3.5. Western Blotting

Cells from different experimental conditions were lysed with ice-cold RIPA lysis buffer as described previously [43]. Protein concentration was determined by a BCA protein assay kit according to the manufacturer’s instructions. Samples with equal amounts of proteins were separated on 9% polyacrylamide gels, transferred to PVDF membrane, and probed with antibodies against phospho-eNOS (Ser1177), phospho-Akt (Ser473), phospho-ERK1/2, respectively. Blots were stripped and reprobed with antibodies for the respective total protein of eNOS, Akt and ERK, where anti-β-actin antibody was used as a loading control.

### 3.6. Mitochondrial Membrane Potential Determination

Mitochondrial membrane potential was analyzed by using a fluorescent dye JC-1 (BestBio, Shanghai China). JC-1 penetrates live cells and healthy mitochondria. At low membrane potentials (apoptotic cells), JC-1 exists as a monomer which emits green fluorescence. JC-1 aggregates and emits red fluorescence at higher membrane potentials (non-apoptotic cells). Experiment was initiated by incubating RGC-5 cells with JC-1 (5 mg/L) for 20 min at 37 °C in the dark and the fluorescence of separated cells was captured by inverted fluorescence microscope (Olympus, Tokyo, Japan), at the wavelengths of 490 nm excitation and 530 nm emission for green; and at 540 nm excitation and 590 nm emission for red. The ratios of red/green fluorescence were calculated.

### 3.7. Measurement of Intracellular ROS Generation

ROS level was evaluated using 2′,7′-dichlorodihydrofluorescin diacetate (DCFH-DA) (Sigma–Aldrich, St. Louis, MO, USA). A membrane-permeable probe that is deesterified intracellularly. The non-fluorescent dye penetrates cells freely and then is hydrolysed to DCFH by intracellular esterases. The DCFH is then trapped inside the cells. Upon oxidation by ROS, DCFH yields the highly fluorescent product dichlorofluorescein (DCF). Treated cells were loaded with DCFH-DA (50 mM as final concentration) in RMPI-1640 media for 30 min in the dark. The cells were rinsed twice with 1× PBS solution and the fluorescence from the DCF was analyzed using a high content screening system (ArrayScanVTI, Thermo Fisher Scientific, Walsam, MA, USA) with the excitation wavelength set at 488 nm and the emission wavelength set at 525 nm. ROS level determined by this method included the H_2_O_2_ residues inside the cells.

### 3.8. Estimation of Lipid Peroxidation

Malondialdehyde (MDA) reacts with thiobarbituric acid (TBA) to produce a fluorescent product. The level of MDA was measured in RGC-5 cells lysates with a microplate reader at a wavelength of 535 nm. RGC-5 cells were treated with GA for 2 h before exposed to H_2_O_2_ and left to grow to more than 90% confluence in 6-well plates. Cells were harvested and washed with PBS after 24 h. The MDA was measured using protocol described in the MDA detection kit from Beyotime Institute of Biotechnology, Nanjing, China.

### 3.9. Determination of NOS Activity

RGC-5 cells were cultured in DMEM with 1% FBS for 24 h following the treatments of H_2_O_2_ (100 µM), GA (10 µM) for 24 h, respectively; or treatment with GA (10 µM) for 2 h before treatment of H_2_O_2_ (100 µM) for another 24 h. The cells without the addition of either H_2_O_2_ or GA were set as control. The medium were removed and the adherent cells were washed with PBS for 1–2 times. Afterwards, the cells were digested by trypsin and passaged into an EP tube. PBS was added to wash the cells. The supernatants were discarded to remove trypsin by low-speed centrifugation. PBS (300 µL) were added to each EP tubes. The cells were disrupted by ultrasonic radiation (power: 300 W; ultrasonic time: 3–5 s) for 4 times at an interval of 30 s to 1 min. The temperature was maintained at 0–5 °C by ice-water bath during the whole ultrasonic process. NOS activities of the cells were assayed using the Typed Nitric Oxide Synthase (NOS) Detection Kit (A014) purchased from Institute of Nanjing Jiancheng Bioengineering according to the manufacturer’s instructions.

### 3.10. Data Analysis and Statistics

All results are reported as means ± SEM for 3–5 experiments. Differences between groups were analyzed using ANOVA, followed by Dunnett’s multi-comparison test with PASW Software (SPSS Inc., Chicago, IL, USA). *p* values < 0.05 were considered statistically significant.

## 4. Conclusions

In short, gardenamide A (GA) protects the rat retinal ganglion (RGC-5) cells against cell apoptosis induced by H_2_O_2_. The protective effect of GA was completely abrogated by the specific phosphoinositide 3-kinase (PI3K) inhibitor LY294002, and the specific protein kinase B (Akt) inhibitor Akt VIII, respectively, indicating that the protective mechanism of GA is mediated by the PI3K/Akt signaling pathway. The specific extracellular signal-regulated kinase (ERK1/2) inhibitor PD98059 could not block the neuroprotection of GA. GA attenuated the levels of reactive oxygen species (ROS) and malondialdehyde (MDA) induced by H_2_O_2_. Western blotting showed that GA promoted the phosphorylation of ERK1/2, Akt and endothelial nitric oxide synthase (eNOS), respectively, and effectively reversed the H_2_O_2_-inhibited phosphorylation of these three proteins. LY294002 completely inhibited the GA-activated phosphorylation of Akt, while only partially inhibiting eNOS. This evidence implies that eNOS may be activated directly by GA. PD98059 attenuated only partially the GA-induced phosphorylation of ERK1/2 with/without the presence of H_2_O_2_, indicating that GA may activate ERK1/2 directly. All these results put together confirm that GA protects RGC-5 cells from H_2_O_2_ insults via the activation of the PI3K/Akt/eNOS signaling pathway. Whether the ERK1/2 signaling pathway is involved requires further investigations.
